# The EIF2α-PERK Signaling Pathway Mediates Manganese Exposure-Induced A1-Type Astrocytes Activation via Endoplasmic Reticulum Stress

**DOI:** 10.3390/toxics13110910

**Published:** 2025-10-23

**Authors:** Jing Wang, Tingting Guo, Yang Hu, Congcong Zhuang, Peng Su, Xinqin Liu

**Affiliations:** Department of Occupational & Environmental Health, The Ministry of Education Key Lab of Hazard Assessment and Control in Special Operational Environment, School of Public Health, Fourth Military Medical University, Xi’an 710032, China; wj941229@163.com (J.W.); xiaoguoting1988@163.com (T.G.); huyang0928@fmmu.edu.cn (Y.H.); 13644843984@163.com (C.Z.)

**Keywords:** manganese, neuroinflammation, astrocyte, mitochondria, PERK

## Abstract

Elevated exposure to manganese (Mn) has been linked to a broad spectrum of neurological disorders, including motor dysfunction. Neuroinflammation with excessively activated astrocytes plays a critical role in the pathogenesis and progression of neurodegenerative diseases. Astrocyte-mediated neuroinflammation plays a dual role due to distinct astrocyte phenotypes, including deleterious A1 and neuroprotective A2. Our previous studies have confirmed that Mn induces activation of astrocytes in the central nervous system, and endoplasmic reticulum (ER) stress has been verified to regulate A1 activation; however, the molecular mechanisms underlying Mn-induced neurotoxicity remain incompletely understood. We establish in vivo and in vitro Mn exposure models and observed that Mn induced A1 activation of astrocytes in both models, with upregulation of A1-specific markers. Sub-cellular morphological analysis showed Mn-induced ER stress in A1-type astrocytes. We found that EIF2α-PERK signaling pathways are activated in astrocytes and drive ER stress and mitochondrial impairment. Suppression of astrocytic PERK, using either ISRIB or GSK2606414, alleviates Mn-induced ER stress and A1 activation, which in turn mitigates the motor deficits induced by Mn exposure. These findings reveal that inhibition of PERK can ameliorate Mn-induced neurotoxicity by suppressing astrocyte activation and preserving organelle homeostasis, offering a potential therapeutic strategy to mitigate the harmful effects of Mn toxicity.

## 1. Introduction

Manganese (Mn) is an essential trace element required for normal growth, development, and intracellular homeostasis [1]. However, chronic exposure to elevated levels of Mn from occupational or environmental sources can result in its accumulation within specific brain regions, particularly the basal ganglia, striatum, and substantia nigra (SN) [2]. Excessive Mn accumulation in the brain has been shown to cause movement disorders in adults that resemble Parkinson’s disease (PD), including tremor, dystonia, abnormal gait, and bradykinesia [3]. Experimental mice exposed to high levels of Mn (100 mg/kg, daily for 2 weeks) similarly exhibit motor deficits and impaired coordination, accompanied by nigrostriatal dopaminergic dysfunction [4,5]. This accumulating evidence emphasizes the significant involvement of Mn in the onset of motor dysfunction and underscores the necessity of clarifying the mechanisms responsible for Mn-induced neurotoxicity to advance the development of effective therapeutic approaches. Our previous research has shown that Mn exposure elicits oxidative stress, inflammatory responses, apoptosis, excitotoxicity, and disturbances in autophagic regulation across different neural cell types [6,7]. The suppression of neuroinflammation has emerged as a potential strategy for mitigating Mn-induced neurotoxic effects [5]. Astrocytes and microglia are critical for Mn-induced neuroinflammation [8]. Nevertheless, the specific role and molecular mechanisms of astrocytes in Mn-induced neurotoxicity remain to be fully elucidated.

Astrocytes are among the most abundant cell populations in the central nervous system (CNS), and play pivotal supportive roles in CNS functions, including neurotransmitter homeostasis, blood–brain barrier integrity, and synaptic development and plasticity [9,10]. Excessive astrocyte reactivity has been implicated in the pathogenesis of a wide range of CNS diseases [11,12]. Recent studies identified two different reactive astrocyte states in response to stimulus, A1 and A2, respectively [13]. Upon stimulation with lipopolysaccharide (LPS), astrocytes transfer into the A1 phenotype, which exhibits neurotoxic properties. The A1 phenotype is characterized by several key markers, including *H2-T23*, *H2-D1*, *Gbp2*, *Psmb8*, and *Srgn*, which contribute to deterioration of neuronal synaptic structures and impairment of neuronal functions. Conversely, A2-phenotype astrocytes are identified by markers such as *Clcf1*, *Tgm1*, *Ptx3*, *S100a10*, and *Emp1*. The A2-phenotype astrocytes demonstrate enhanced expression of multiple neurotrophic factors, facilitate synaptic regeneration in neurons, and primarily serve a neuroprotective function within the nervous system [14,15]. Nevertheless, the precise role and significance of A1 astrocytes in the pathogenesis and treatment of Mn-induced neurotoxicity remain unclear.

Mounting evidence indicates that dysfunction of key cellular organelles, particularly the endoplasmic reticulum (ER) and mitochondria, contributes substantially to stress-induced pathophysiological alterations [16]. Clinical studies have further demonstrated significant associations between ER stress, mitochondrial impairment, and psychiatric disorders, with particular relevance to major depressive disorder [17,18]. Importantly, emerging research highlights the critical role of mitochondria-associated ER membranes’ (MAMs) specialized organelle contact sites that coordinate essential cellular processes including calcium homeostasis, mitochondrial dynamics, autophagy, and programmed cell death [19,20,21]. Protein kinase RNA-like endoplasmic reticulum kinase (PERK), an ER stress sensor, is activated in response to the accumulation of misfolded proteins [22]. During ER stress, PERK activation triggers mitochondrial fusion, preventing premature apoptotic fragmentation of the mitochondria in astrocytes [23]. It remains unclear whether PERK-mediated ER stress leads to astrocyte activation after Mn exposure.

Thus, building on previous studies, we sought to investigate whether the PERK pathway contributes to Mn-induced mitochondrial dysfunction, astrocytic A1 activation, and subsequent dopaminergic neuronal injury. Our findings revealed that Mn-induced A1 astrocytic activation was mediated by mitochondrial damage and enhanced PERK signaling. Moreover, pharmacological inhibition of PERK with ISRIB effectively abolished Mn-induced ER stress, neurotoxicity, and mitochondrial abnormalities in mice brains. These findings suggest a potential therapeutic treatment in Mn neurotoxicity, and supplied data support on the mechanism of PD.

## 2. Materials and Methods

### 2.1. Mice and Treatment

For all experiments, male C57BL/6 mice (4–6 weeks old) were obtained from the Fourth Military Medical University (Xi’an, Shaanxi, China). The mice were housed under standard conditions at 23 ± 2 °C with a 12 h light/dark cycle (lights on at 7:00 A.M.) and provided with a normal diet and water ad libitum. After a one-week acclimatization period, the animals were randomly assigned to two experimental groups, a control group (Con) receiving subcutaneous injections of 0.9% saline, and treatment group (Mn) administered MnCl_2_·4H_2_O (Sigma, Burlington, MA) (100 mg/kg) via the same route. Injections were performed on days 1, 4, 7, 10, and 13, following an established protocol [3]. Upon completion of the experimental procedures, all mice were euthanized with an overdose of sodium pentobarbital (Sigma-Aldrich, St. Louis, MO, USA), (150 mg/kg, i.p.), followed by cervical dislocation to ensure death. All animal experiments were conducted in strict accordance with the ethical guidelines approved by the Institutional Animal Care and Use Committee of Air Force Medical University (Approval No. 202003-201).

### 2.2. Quantification of Mn in Blood and Brain Tissue

The blood samples were obtained by cardiac puncture from anesthetized mice. After blood collection, the animals were perfused transcranial with saline and then with 4% paraformaldehyde (Macklin, Shanghai, China). The cerebral cortex was subsequently isolated. For blood Mn analysis, 100 μL aliquots were digested in 3.9 mL of 0.5 N nitric acid (Sinopharm Chemical Reagent Co., Ltd., Shanghai, China)containing 0.01% Triton X-100 (Sigma, Burlington, MA, USA), vertexed for 10 s, and centrifuged (10 min at 7500 r/min at room temperature); the supernatant was then collected for assay. The cerebral cortex tissues were homogenized in a solution 0.5 N nitric acid, 0.5 N perchloric acid (Sinopharm Chemical Reagent Co., Ltd., Shanghai, China), and 0.01% Triton X-100 to achieve a 1:10 dilution. All tubes were pre-cleaned with nitric acid and verified to be lead-free.

Measurements were performed using graphite furnace atomic absorption spectrometry (PerkinElmer, Waltham, MA, USA). A 20 μL sample aliquot, along with 20 mL of 0.2% magnesium nitrate modifier (Sinopharm Chemical Reagent Co., Ltd., Shanghai, China), was automatically injected into the graphite furnace. The Mn concentration was measured at 283.3 nm using a programmed heating cycle under an argon atmosphere. All samples were analyzed in duplicate, and results were calculated from the mean peak heights. Calibration standards (5, 10, and 20 ppm) were prepared by serial dilution of a 1000 ppm Mn standard. Recovery was assessed by the standard addition method, whereby the standard was added to separate samples to a final concentration of 10 ppm.

### 2.3. Isolated Primary Astrocytes and Primary Neurons

Primary neurons and primary astrocytes were isolated from the cerebral cortices of newborn (P0-P2) C57BL/6 mice according to our previous protocol [24]. Substantia nigra and striatum tissues were mechanically dissociated, and the resulting cell suspension was filtered through a 70 μm (130-098-462, Miltenyi Biotec, Bergisch Gladbach, Germany) nylon mesh to remove meningeal debris. To enhance initial cell adhesion and viability, all culture surfaces were pre-coated with 0.1 mg/mL poly-L-lysine (PLL) (Sigma, Burlington, MA, USA) for 1 h at room temperature, followed by three washes with sterile water. Cells from 4 to 5 littermates were pooled and seeded into PLL-coated T-225 flasks (Corning, Corning, NY, USA) in high-glucose Dulbecco’s Modified Eagle Medium (DMEM) (Gibco, NY, USA) supplemented with 10% heat-inactivated fetal bovine serum (Gibco, Grand Island, NY, USA), 2 mM glutamine (Gibco, NY, USA), 100 U/mL penicillin (Thermo Fisher Scientific, Grand Island, NY, USA), and 100 μg/mL streptomycin (GIBCO, Billings, MT, USA). The medium was replaced 24 h post-seeding to remove non-adherent cellular cell debris.

To separate astrocytes from other glial cells, we employed a differential adhesion protocol. After 10–14 days in vitro culture, microglia and oligodendrocyte precursors were detached by subjecting the flasks to orbital shaking at 110 rpm for 1 h at 37 °C. This process yields two distinct fractions: (1) the supernatant, which contains the dislodged microglia (and other loosely adherent cells); (2) the adherent monolayer, which constitutes the astrocyte-enriched fraction.

### 2.4. Western Blot Analysis

Brain tissues from mice exposed to different concentrations of Mn were collected, placed in 1.5 mL EP tubes, and washed three times with PBS. Then, samples were lysed in ice-cold RIPA buffer containing protease inhibitor cocktail, followed by centrifugation at 12,000× *g* for 10 min at 4 °C. Supernatants were transferred to fresh 1.5 mL EP tubes, and protein concentrations were determined using a BCA assay kit (Thermo, Cat#23225, Waltham, MA, USA).

Equal amounts of protein were resolved by SDS-PAGE and transferred onto polyvinylidene fluoride (PVDF) membranes. After blocking with 5% BSA, membranes were incubated overnight at 4 °C with primary antibodies against PERK and p-PERK (Santa Cruz, Dallas, TX, USA), EIF2α (Proteintech, Wuhan, Hubei, China) and p-EIF2α (Cell Signaling Technology, Danvers, MA, USA), and ATF4 (Proteintech, Wuhan, Hubei, China). Following TBST washes, membranes were incubated with HRP-conjugated secondary antibodies for 1 h at room temperature. Protein bands were visualized using a chemiluminescence detection system (Fusion Solo6S, VILBER, Eberhardzell, Germany) and quantified with ImageJ software (Version 1.54p). Each biological replicate represents cells harvested from an independent primary culture preparation, or “Each *n* represents one mouse”, “*n* = 4 biologically independent mice per group”, or “*n* = 3 independent cell culture experiments”.

### 2.5. Immunofluorescence

Fixed and snap-frozen mouse brain tissues were coronally sectioned at the substantia nigra (−2.70 to −3.40 mm from bregma) and striatum (+0.98 to +1.18 mm from bregma) at a thickness of 20 μm. Immunofluorescence was performed to detect TH (abcam, Cat#137869, Waltham, MA, USA), C3 (abcam, Cat#200999), S100β (abcam, Cat#52642), SNAP25 (abcam, Cat#109105), PSD95(abcam, Cat#238135), and GFAP (abcam, Cat#7260) using tissue sections from three mice per group. Sections mounted on glass slides were washed twice with PBST (1 × PBS, 0.3% Triton X-100) and incubated in blocking buffer (10% normal goat serum, 1% bovine serum albumin, and 0.3% Triton X-100 in 1 × PBS) for 1 h at room temperature. Primary antibodies against TH, C3, S100β, SNAP25, PSD95, and GAFP (1:250 in the blocking buffer) were applied overnight at 4 °C in a dark humidity chamber. After washing, sections were incubated for 2 h at room temperature with secondary antibodies conjugated to Alexa Fluor 488, 568, and/or 647 (1:1000) (Thermo). Slides were washed, air-dried, and mounted with coverslips. Fluorescence intensity from the same anatomical regions was evaluated using a Ts2R fluorescence microscope (Nikon Instruments, Tokyo, Japan) and a SPEII confocal microscope (Leica Microsystems Inc., Wetzlar, Germany). Each biological replicate represents one mouse, “*n* = 5 biologically independent mice per group”.

### 2.6. Quantitative RT-PCR

Total RNA was extracted from [tissue/cells] using an RNeasy Mini Kit (QIAGEN, Cat#74104, Venlo, The Netherlands) according to the manufacturer’s instructions. RNA purity and concentration were assessed with a NanoDrop 8000 spectrophotometer (Thermo Fisher, Wilmington, DE, USA), and integrity was verified by agarose gel electrophoresis. Only samples with A260/280 ratios of 1.9–2.1 were used.

For two-step assays, cDNA was synthesized from 500 ng^–1^ μg RNA using a reverse transcription kit (Thermo Fisher) with random hexamers and oligo(dT) primers, followed by qPCR using a SYBR Green Master Mix on a Bio-Rad (Hercules, CA, USA). Reactions (10 μL) included 1 × SYBR mix and 200–400 nM forward/reverse primers. Thermal cycling was carried out at 95 °C for 2 min, followed by 40 cycles of 95 °C for 15 s and 60 °C for 30 s, with melting curve analysis performed to verify specificity. Primer efficiency was confirmed to be 90–110% (R^2^ ≥ 0.99). For detailed primer sequences, see Table S1.

Each sample was run in triplicate. No-template controls and no-reverse-transcriptase controls were included. Relative gene expression was normalized to β-Actin and calculated using the 2^−ΔΔCt^ method. Results are presented as mean ± SEM from at least three biological replicates. Each biological replicate represents cells harvested from an independent primary culture preparation, or “Each n represents one mouse”, “*n* = 3 biologically independent mice per group”, or “*n* = 3 independent cell culture experiments”. Each sample was run in duplicate wells for qPCR analysis.

### 2.7. Open-Field Test (OFT)

Open-field testing was performed to evaluate locomotor activity. Mice were placed individually in a plastic chamber (50 × 50 × 60 cm) with a central zone measuring 40 × 40 cm and allowed to freely explore for 5 min. Behavioral trajectories were recorded, and the total distance traveled was quantified using EthoVision XT software (version 11.5). Locomotor activity was assessed based on the total distance moved and the frequency of entries into the central zone. Each biological replicate represents one mouse, “*n* = 10 biologically independent mice per group”.

### 2.8. Rotor Experiment

Rotarod testing was performed to evaluate motor coordination and balance. Prior to testing, mice were habituated to the apparatus. The rotating rod (30 cm in diameter) was set to an initial speed of 10 rpm, and mice were placed individually on the rod to maintain balance. The rotation speed was gradually increased to 30 rpm, and the latency to fall was recorded for each mouse. Each mouse underwent three trials, and the mean latency was calculated. Each biological replicate represents one mouse, “*n* = 10 biologically independent mice per group”.

### 2.9. Pole Climbing Experiment

Mice were acclimatized to the apparatus during a 24 h training period preceding testing. Following a 24 h habituation phase, mice performed a pole-climbing task on a 50 cm vertical rod. The primary outcome measure was descent latency (time to climb from top to bottom), assessed over three trials per subject with mean values computed for statistical analysis. Each biological replicate represents one mouse, “*n* = 10 biologically independent mice per group”.

### 2.10. Transmission Electron Microscopy Analysis

Mitochondrial ultra-structure was examined by transmission electron microscopy (TEM). Astrocytes were fixed with ice-cold 2.5% glutaraldehyde in 0.1 M cacodylate buffer (pH 7.4) for 30 min at 4 °C, followed by post-fixation in 1% osmium tetroxide (OsO_4_) in the same buffer. After dehydration through a graded ethanol series, samples were embedded in Epon 812 epoxy resin. Ultrathin sections (70–80 nm) were cut using a diamond knife, double-stained with uranyl acetate and lead citrate, and examined with a H-600 transmission electron microscope (Hitachi Ltd., Tokyo, Japan) at an accelerating voltage of 80 kV.

### 2.11. MitoTracker Green Staining

Mitochondrial morphology was assessed using MitoTracker™ Green FM (Thermo Fisher Scientific). The stock solution was diluted in culture medium to a final concentration of 50 nM. Cells were incubated with 1 mL staining solution at 37 °C for 30 min, followed by washing with fresh medium. Mitochondrial morphology was visualized and imaged using a laser confocal microscopy (Nikon Instruments). Each biological replicate represents one mouse, “*n* = 5 biologically independent mice per group”.

### 2.12. Statistical Analysis

All experiments were repeated at least three times. Data are presented as mean ± SEM. Statistical analyses were performed using SPSS software (Version 26.0, IBM SPSS Statistic, Armonk, NY, USA). Relative transcript levels from RT-qPCR were calculated using the 2^−ΔΔCt^ method. Comparisons between two groups were analyzed with a two-tailed Student’s *t*-test, whereas multiple group comparisons were evaluated using two-way ANOVA followed by Tukey’s post hoc test. A *p* value < 0.05 was considered statistically significant.

The minimum sample size for each group was *n* = 3, determined based on prior studies and preliminary data to ensure sufficient power to detect biologically relevant differences. No statistical methods were used to predetermine sample size; however, group sizes were consistent with those generally employed in the field. Power analysis indicated that the chosen sample sizes provided at least 80% power to detect an effect size of 20–25% change at α = 0.05.

## 3. Results

### 3.1. Mn Exposure Led to Impairment of Dopaminergic Neurons In Vivo and In Vitro

**Figure 1:**
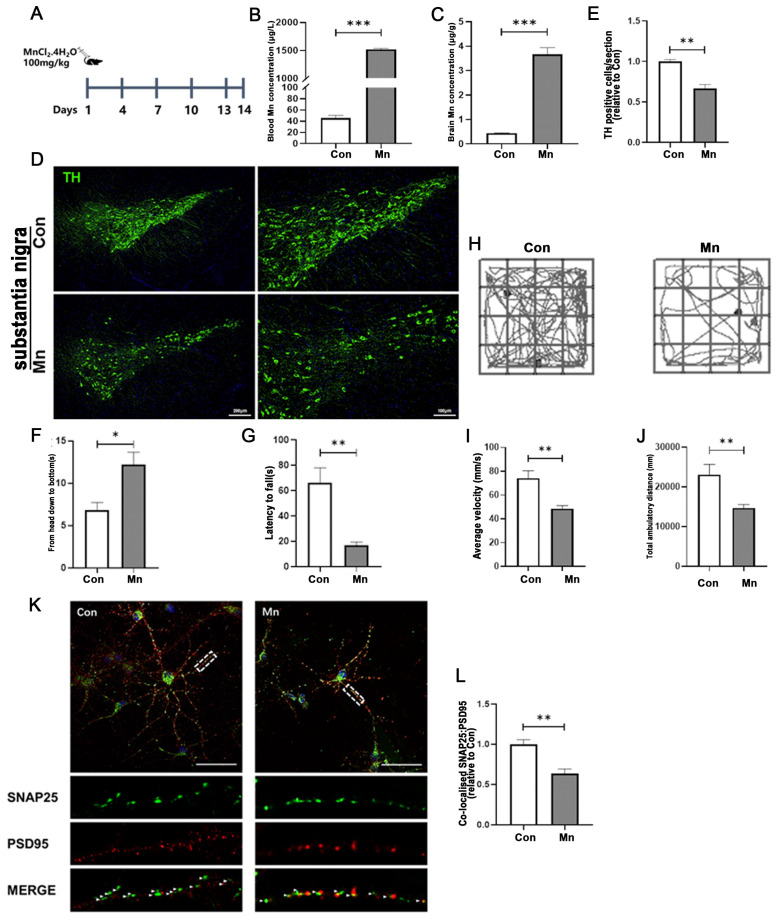
Mn-induced motor impairment and locomotor deficits. (**A**) Flowchart illustrating the establishment of a mouse Mn exposure model via subcutaneous injection. (**B**,**C**) Atomic absorption spectroscopy analysis of Mn levels in (**B**) blood and (**C**) brain of Mn-exposed mice. (**B**) Blood Mn levels in the Mn-exposed mice (*n* = 5, *** *p* < 0.001 vs. Con). (**C**) Brain Mn levels in Mn-exposed mice (*n* = 5, *** *p* < 0.001 vs. Con). (**D**) Representative immunofluorescence staining of tyrosine hydroxylase (TH) in coronal sections of the substantia nigra after Mn exposure, as described in the experimental procedures. Scale bar: left side, 200 μm; right, 100 μm. (**E**) Quantification of TH fluorescence intensity across treatment groups (*n* = 10, ** *p* < 0.01 vs. Con). (**F**) Pole test: time required for mice to climb down the pole (*n* = 10, * *p* < 0.05 vs. Con). (**G**) Rotarod test: latency to fall from the fatigue apparatus (*n* = 10, ** *p* < 0.01 vs. Con). (**H**–**J**) Open-field test evaluating locomotor activity after Mn exposure (**H**), representative movement traces, (**I**) average walking velocity, and (**J**) total ambulatory distance (*n* = 10, ** *p* < 0.01 vs. Con). (**K**) Immunofluorescence staining showing Mn-induced activation of A1-activition astrocytes and their impact on primary neuronal synapses. Synaptic markers PSD95 (red) and SNAP25 (green) were visualized to evaluate colocalization., Scale bar = 50 μm. Enlarged views of boxed areas are shown below. (**L**) Quantitative analysis of SNAP25 and PSD95 colocalization in immunofluorescence images (*n* = 3, ** *p* < 0.01 vs. Con among treatment groups(ACM)).

Exposure to excessive manganese (Mn) is associated with neuroinflammation and extrapyramidal motor deficits, which resemble the features of Parkinson’s disease. To define the neurotoxic effects of Mn exposure on motor coordination and dopaminergic neurons in the basal ganglia of mice, we utilized a subacute manganese-exposed mouse model (Figure 1A). Mn concentrations were markedly elevated in both in blood and brain tissues. As shown in (Figure 1B,C), Mn levels in the blood and brain were significantly increased after 14 days of Mn injection (*p* < 0.001). To further corroborate the neurological damage induced by Mn and explore the underlying mechanism of Mn movement dysfunction, we performed TH staining following Mn exposure. Mn-treated mice exhibited a significant reduction in the number of dopaminergic neurons in both the substantia nigra (SN) and striatum (Figure 1D,E and Figure S1A,B).

To a certain extent, the behavior of mice reflect the normal functioning of the nervous system. Therefore, motor behavior was examined at 14 days of Mn exposure. We used three behavior experiments to investigate the effects of manganese exposure on motor coordination and balance in mice. Motor coordination and balance in mice exhibited pronounced motor dysfunction in pole climbing, alongside a notable reduction in dopamine neurons within the substantia nigra compacta. Mice exposed to Mn took significantly longer to complete the pole climbing task than the control group (Figure 1F). The rotarod test was utilized to evaluate motor coordination in rodents. The results indicated that after Mn exposure the amount of time mice spent on the rotarod was significantly shorter than that of the control group (Figure 1G). Mn-treated mice showed significantly decreased average velocity during the removal test compared to controls. These results showed that movement distance and velocity significantly decreased with Mn exposure by the open-field test (Figure 1H–J). Collectively, the results of the three behavioral experiments demonstrated that subcutaneous injection of manganese significantly impaired the motor coordination abilities of the mouse model.

To further investigate the detailed effect between Mn-induced neurotoxicity and astrocyte A1 activation, we performed in vitro cellular experiments (Figure S1C). Astrocyte cultures stimulated with Mn and which had developed A1 activation were transferred to neuronal medium for co-culture. We investigated the impact of Mn-induced A1-reactive astrocytes on synapses in primary neurons. Mn-exposure-induced A1-reactive astrocytes did not significantly increase the number of primary neuronal deaths (Figure S1D,E). SNAP25 and PSD95 were utilized to label the presynaptic and postsynaptic membranes, respectively. Compared to the control group, manganese exposure led to a significant reduction in the number of synapses in primary neurons. (Figure 1I,J) (*p* < 0.01).

### 3.2. Manganese Exposure Triggers A1 Activation of Astrocytes In Vivo and In Vitro

**Figure 2:**
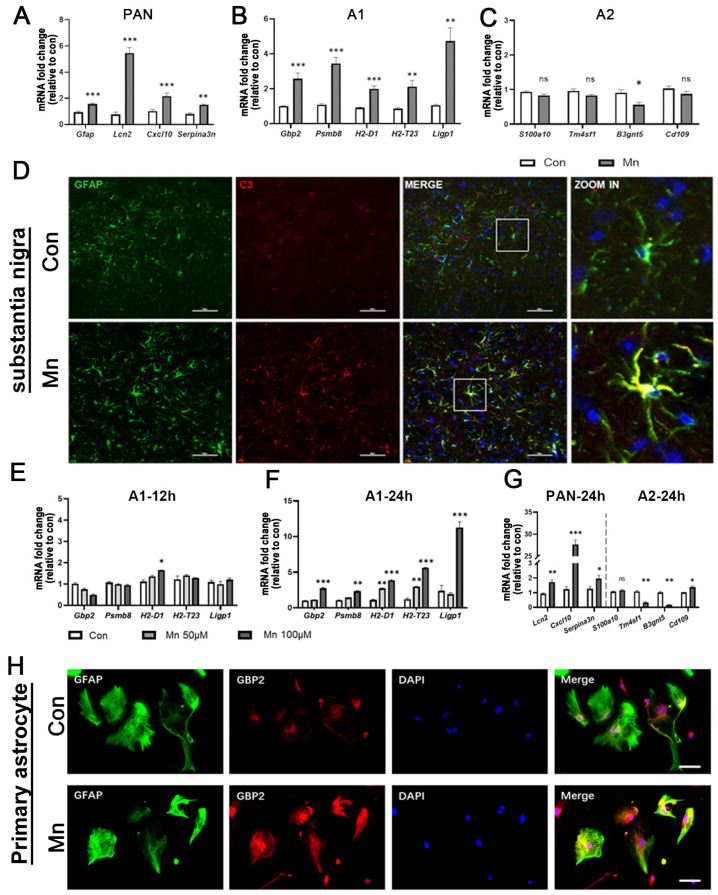
Exposure to Mn induces activation of astrocytes in the SN and in cultured primary astrocytes. (**A**–**C**) Bar charts showing relative expression of (**A**) PAN-specific, (**B**) A1-specific, and (**C**) A2-specific (**C**) transcripts in the SN, as analyzed by RT-qPCR (*n* = 5, * *p* < 0.05 vs. Con, ** *p* < 0.01 vs. Con, *** *p* < 0.001 vs. Con, ns, *p* > 0.05 vs. Con). (**D**) Representative immunofluorescence staining of S100β and C3 proteins in coronal sections of the SN after Mn exposure, as described in the experimental procedures. Scale bar = 50 μm. Labeling indicates GFAP (green) and C3 (red) proteins. The area within the white box is enlarged on the right. (**E**,**F**) Relative mRNA expression of A1-special transcripts in primary astrocytes exposed to 50 or 100 μM Mn for 12 h and 24 h (*n* = 5, * *p* < 0.05 vs. Con, ** *p* < 0.01 vs. Con, *** *p* < 0.001 vs. Con). (**G**) Relative mRNA expression of PAN-specific and A2-specific transcripts in primary astrocytes exposed to 100 μM of Mn for 24 h (*n* = 5, * *p* < 0.05 vs. Con, ** *p* < 0.01 vs. Con, *** *p* < 0.001 vs. Con, ns *p* > 0.05 vs. Con). (**H**) Representative immunofluorescence staining of primary astrocytes, showing GBP2 (red), GFAP (green), and DAPI (blue). Scale bar = 50 μm.

Previous studies have demonstrated that astrocytes are a primary target of Mn neurotoxicity, as Mn preferentially accumulates in these cells. However, the mechanisms underlying Mn-induced astrocyte dysfunction and its role in metal neurotoxicity remain incompletely defined. Our study systematically interrogated the morphological, molecular, and functional perturbations of dopaminergic neurons and astrocytes within the SN and striatum, which are characterized by prominent Mn sequestration. Mn exposure markedly upregulated astrocyte activation-related genes across multiple brain regions (Figure 2A–C and Figure S2A–C). To determine the effects of Mn exposure on astrocyte subtypes, we quantified mRNA expression levels of PAN-specific, A1-specific, and A2-specific markers. Transcripts of PAN-specific markers (*Gfap*, *Lcn2*, *Cxcl10*, and *Serpin2a*) were increased in the SN and striatum of Mn-exposed mice (Figure 2A and Figure S2A, *p* < 0.001). mRNA level of A1-specific markers (*Gbp2*, *Ligp1*, *Psmb8*, *H2-D1*, and *H2-T23*) were increased in the SN and striatum of mice (Figure 2B and Figure S2B, *p* < 0.001). In contrast, the expression of A2-specific markers (*S100a10*, *Tm4sf1*, *B3gnt5*, and *Cd109*) did not show significant changes in either the SN or striatum (Figure 2C and Figure S2C). As C3 is one of the characteristics of A1 astrocytes and reflects A1-activation astrocytes, we next performed co-labeling using S100β and GFAP to identify astrocytes in the SN and striatum. The proportion of A1 astrocytes defined as C3 and GFAP double-positive (C3+GFAP+) cells was increased following Mn exposure (Figure 2D and Figure S2D). Immunofluorescence quantification of GFAP/S100β and C3 in Mn-targeted regions further indicated that Mn exposure enhanced A1-type astrocyte activation in the SN and striatum (Figure S2E,F, *p* < 0.01).

To eliminate interference from other glial cell types and further clarify the effects of Mn exposure on astrocytes, we established an in vitro Mn exposure model. To further explore the underlying mechanisms, we employed primary astrocytes cultures (see Section 2). Astrocyte activation following manganese exposure was evaluated using isolated and cultured primary astrocytes. To determine astrocyte subtype responses, we measured the mRNA expression levels of A1-specific and A2-specific markers after 12 and 24 h of Mn exposure (Figure 2E,F). The transcripts of A1-specific markers (*Gbp2*, *Ligp1*, *Psmb8*, *H2-D1*, and *H2-T23*) were significantly increased at both 12 and 24 h post-Mn exposure (Figure 2E,F). In contrast, the transcripts of PAN-specific markers and A2-specific markers (*S100a10*, *Tm4sf1*, *B3gnt5*, and *Cd109*) were not significantly changed after 12 h. We only showed expression of mRNA for PAN-specific markers, and A2-specific markers showed no significant change at 12 h. The mRNA expression of PAN-specific markers (Lcn2, Cxcl10, and Serpin2a) and A2-specific markers (Tm4sf1, B3gnt5, and Cd109) was significantly upregulated after 24 h of Mn exposure (Figure 2G). As *Gbp2* is a characteristic marker of A1 astrocytes and its expression GBP2 reflects A1 activation, we next used GBP2 and GFAP to co-label the primary astrocytes (Figure 2H). Mn exposure increased GBP2 localization in primary astrocytes (Figure S2G). However, GFAP levels did not significantly increase in the primary astrocytes (Figure S2H).

### 3.3. Structural and Functional Alterations of the Endoplasmic Reticulum and Mitochondria in A1 Astrocytes Induced by Mn Exposure

**Figure 3:**
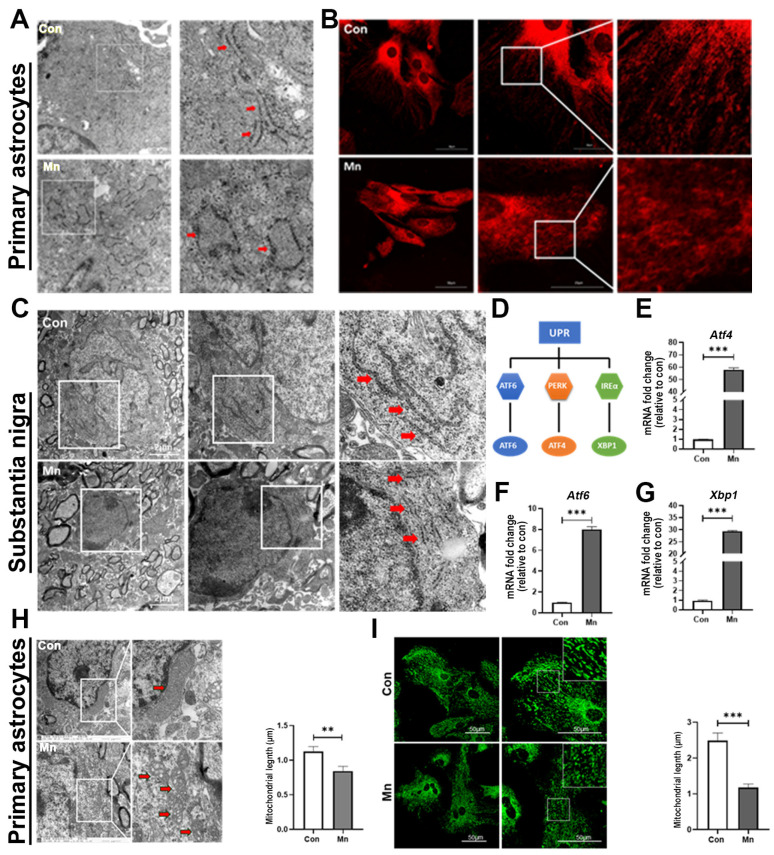
Mn exposure alters mitochondrial and endoplasmic reticulum (ER) structures. (**A**) Representative transmission electron microscopy (TEM) images. Scale bar = 0.5 μm. Enlarged views of boxed areas are shown right, highlighting the endoplasmic reticulum (red arrows). (**B**) Representative fluorescence images of ER tracker red in primary astrocytes (left panel). Scale bars = 50 μm. Enlarged views of boxed areas are shown in the right panel. Scale bar = 25 μm. (**C**) Representative TEM images showing ultrastructural alterations in astrocytes in the SN (red arrowheads indicating ER). Scale bar: left, 2 μm; right, 1 μm. (**D**) Schematic diagram of the unfolded protein response (UPR) signaling pathway. (**E**–**G**) Quantitative RT-qPCR analysis of UPR-specific markers (*Atf4*, *Atf6*, and *Xbp1*) in the primary astrocytes (*** *p* < 0.001 vs. Con). (**H**) Representative TEM images showing mitochondrial alterations in primary astrocytes. Scale bar: left, 2 μm; right, 1 μm. Statistical analysis of mitochondrial length is shown (*n* = 5, ** *p* < 0.01 vs. Con). (**I**) Representative fluorescence images of MitoTracker red in primary astrocytes (left panel). Scale bars: =50 μm. Enlarged views of boxed areas are shown in the right panel. Quantitative analysis of mitochondrial length is provided (*n* = 5, *** *p* < 0.001 vs. Con).

We next investigate the cellular stress responses of A1 astrocytes to Mn stimulation. Primary astrocytes were isolated from postnatal mice (see Section 2). Transmission electron microscopy (TEM) revealed that Mn exposure caused expansion and swelling of the endoplasmic reticulum (ER) in A1 astrocytes (Figure 3A), suggesting that activated astrocytes undergo ER stress. To specifically examine the ER, we employed ER-tracker dye staining. Live-cell imaging demonstrated that Mn exposure induced swelling and structural disorganization of the ER in primary astrocytes (Figure 3B). To further clarify the structural and functional alterations of the ER in A1 astrocytes, we used TEM to analyze ER morphology in the SN and striatum. Astrocytes in the SN and striatum of control mice exhibited an ER with tubular morphology and intact membranes, whereas Mn-exposed astrocytes displayed a swollen ER with widened membrane gaps and aberrant structures (Figure 3C and Figure S3A). Consistent with these findings, RT-PCR of primary astrocytes revealed elevated expression of unfolded protein response (UPR)-related genes, including *Atf4*, *Atf6*, and *Xbp1*, following Mn exposure (Figure 3D–G). The transcript levels of these UPR-specific markers were significantly upregulated (Figure 3E–G).

We next observed whether other organelle were affected by Mn exposure. TEM analysis revealed that Mn exposure caused mitochondria swelling in A1 astrocytes. Furthermore, we observed an increase in mitochondrial number accompanied by a reduction in mitochondrial length in astrocytes following Mn exposure (Figure 3H). MitoTracker staining further demonstrated that, compared with controls, Mn exposure significantly enhanced mitochondrial fragmentation in primary astrocytes (Figure 3I). In summary, these findings suggest that Mn exposure heightens the sensitivity of primary astrocytes and induces ER stress and mitochondrial dysfunction in A1 astrocytes.

### 3.4. PERK Regulates Astrocyte Activation Towards Neurotoxic Phenotype

Our results demonstrated that the mRNA levels of three key transcription factors of the unfolded protein response (UPR) pathway, *Atf4*, *Atf6*, and *Xbp1*, were significantly upregulated following Mn exposure, with *Atf4* showing the most pronounced increase (Figure 3F). Double-stranded RNA-dependent protein kinase-like ER kinase (PERK) is a ubiquitous ER stress-sensing protein in eukaryotic cells. Previous studies have reported that the PERK pathway has been activated by Mn exposure in astrocytes. However, the relationship between PERK pathway activation and the induction of the A1 astrocyte phenotype after manganese exposure remains unclear. We therefore sought to determine whether the significant increase in ATF4 expression was attributable to activation of the PERK pathway in astrocytes following Mn exposure.

**Figure 4:**
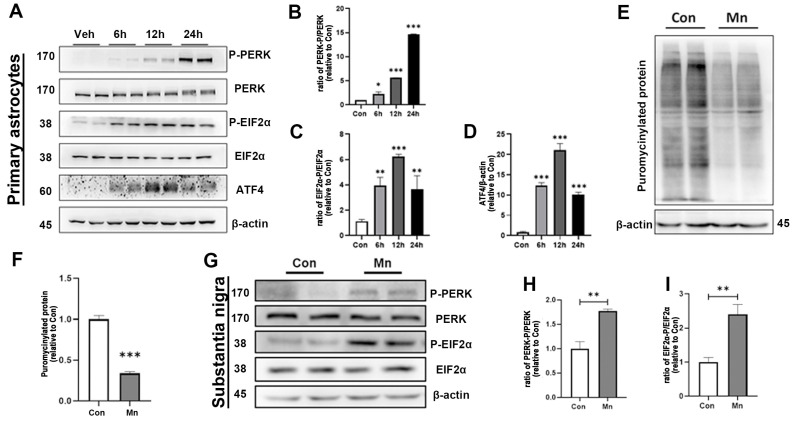
PERK pathway contributes to Mn-induced neurotoxicity in mice and primary astrocytes. (**A**) Western blot analysis of PERK/p-PERK, EIF2α/p-EIF2, and ATF4 protein levels in primary astrocytes after Mn exposure (6 h, 12 h, and 24 h). β-actin was used as loading control (*n* = 4). (**B**) Quantitative grayscale analysis of p-PERK/PERK ratios (* *p* < 0.05 vs. Con, *** *p* < 0.001 vs. Con). (**C**) Quantitative grayscale analysis of p-eIF2α/eIF2α ratios (*n* = 4, ** *p* < 0.01 vs. Con, *** *p* < 0.001 vs. Con). (**D**) Quantitative grayscale analysis of ATF4 levels (*n* = 4, *** *p* < 0.001 vs. Con). (**E**) Global protein synthesis in control and Mn-exposed astrocytes was assessed using a puromycylation assay followed by Western blotting, with β-actin as a reference. (**F**) Quantitative grayscale analysis of puromycinylated proteins (*n* = 4, *** *p* < 0.001 vs. Con). (**G**–**I**) Western blot analysis of protein expression in the substantia nigra (SN) of mouse brains after Mn exposure: (**H**) P-PERK/PERK and (**I**) p-EIF2α/EIF2αratios (*n* = 4, ** *p* < 0.01 vs. Con).

The protein levels of p-PERK, p-EIF2α, and ATF4 were significantly enhanced in astrocytes following Mn exposure (Figure 4A–C). Western blot analysis further demonstrated that the phosphorylation of PERK and EIF2α progressively increased with the prolonged Mn exposure compared with the control group (Figure 4B,C). To assess whether PERK activation influences global protein synthesis, a puromycylation assay was performed to detect puromycin incorporation into ribosome-bound nascent chains (Figure 4E). Mn exposure broadly reduced the de novo protein synthesis (Figure 4F). In addition, the protein ratios of p-PERK/PERK and EIF2α/p-EIF2α were significantly increased in the SN and striatum following Mn exposure (Figure 4G–I and Figure S4A–C). Results showed that Mn exposure increased the p-PERK/PERK ratio in the SN and striatum of control mice, and this effect was further exacerbated in primary astrocytes.

### 3.5. Inhibition of PERK in Primary Astrocytes Protects DA Neurons from Mn-Exposure-Induced A1 Astrocytes Toxicity

**Figure 5:**
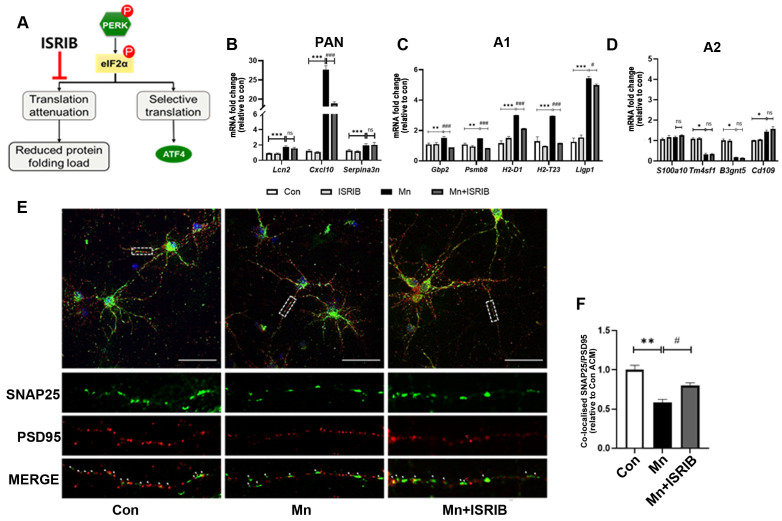
Inhibition of PERK signaling alleviates astrocyte activation and neurotoxicity. (**A**) Schematic diagram illustrating inhibition of the PERK pathway by ISRIB. (**B**–**D**) Bar charts showing relative expression of (**B**) PAN-specific, (**C**) A1-specific, and (**D**) A2-specific transcripts in primary astrocytes, analyzed by RT-qPCR. The experiment included four groups: Con, ISRIB, Mn, and Mn+ ISRIB (*n* = 3, * *p* < 0.05 vs. Con, ** *p* < 0.01 vs. Con, *** *p* < 0.001 vs. Con, ^#^ *p* < 0.05 vs. Mn, ^###^ *p* < 0.01 vs. Mn, ns *p* > 0.05 vs. Mn). (**E**) Representative immunofluorescence staining showing the effect of Mn-induced A1-activation astrocytes on primary neuronal synapses. Synapse markers PSD95 (red) and SNAP25 (green) were visualized to evaluate colocalization., Scale bar = 50 μm. Enlarged views of boxed areas are shown below. (**F**) Quantitative analysis of SNAP25 and PSD95 colocalization in immunofluorescence images (*n* = 3, ** *p* < 0.01 vs. Con ACM, ^#^ *p* < 0.05 vs. Mn ACM).

To further validate the role of the PERK pathway in Mn-induced A1 astrocyte activation, we employed the PERK inhibitor ISRIB to selectively block the PERK pathway and subsequently examined their effects on A1 astrocyte activation (Figure 5A and Figure S5A–C). Importantly, inhibition of PERK signaling with the downstream inhibitor ISRIB significantly reduced the mRNA expression of A1-specific markers (*Gbp2*, *Ligp1*, *Psmb8*, *H2-D1*, and *H2-T23*) (Figure S5F). No significant changes in expression of PAN-specific and A2-specific markers were observed in either the Mn-treated or Mn + ISRIB treated groups (Figure 5B,D). In conclusion, both PERK inhibitors partially alleviated A1 astrocyte activation induced by Mn exposure.

Previous studies have shown that neurotoxic A1 astrocytes can damage the neuronal synaptic structure, but whether the PERK pathway is a key mechanism mediating this neurotoxicity remains unclear. To address this, we performed conditioned culture experiments using primary neurons. Synaptic integrity was assessed in control (Con), Mn-treated, and Mn+ISRIB-treated groups by immunolabeling with anti-SNAP25 and anti-PSD95 antibodies to mark presynaptic and postsynaptic membranes, respectively (Figure 5E). The total number of synapses was quantitated for each experimental group. Notably, the Mn+ISRIB group exhibited a significant increase in synapse number compared with the Mn group (Figure 5F, *p* < 0.05). These findings support our hypothesis that the activation of the PERK pathway is a key mechanism by which Mn exposure induces A1 astrocyte activation and subsequent neurotoxicity.

To further validate whether PERK is involved in the regulatory mechanism of Mn-induced A1 astrocyte activation, we employed another inhibitor GSK2606414, which directly binds to the PERK kinase domain and inhibits PERK activation by downregulating its autophosphorylation (Figure S5). Western blot analysis and RT-qPCR results of GSK260641 were consistent with data of ISRIB. These results indicate that PERK inhibition partially suppresses Mn-induced A1 activation in primary astrocytes, suggesting that activation of the PERK pathway is an important mechanism underlying Mn-induced A1 activation.

### 3.6. Inhibition of PERK Partially Suppresses Astrocyte A1 Activation and Attenuates the Motor Deficits

**Figure 6:**
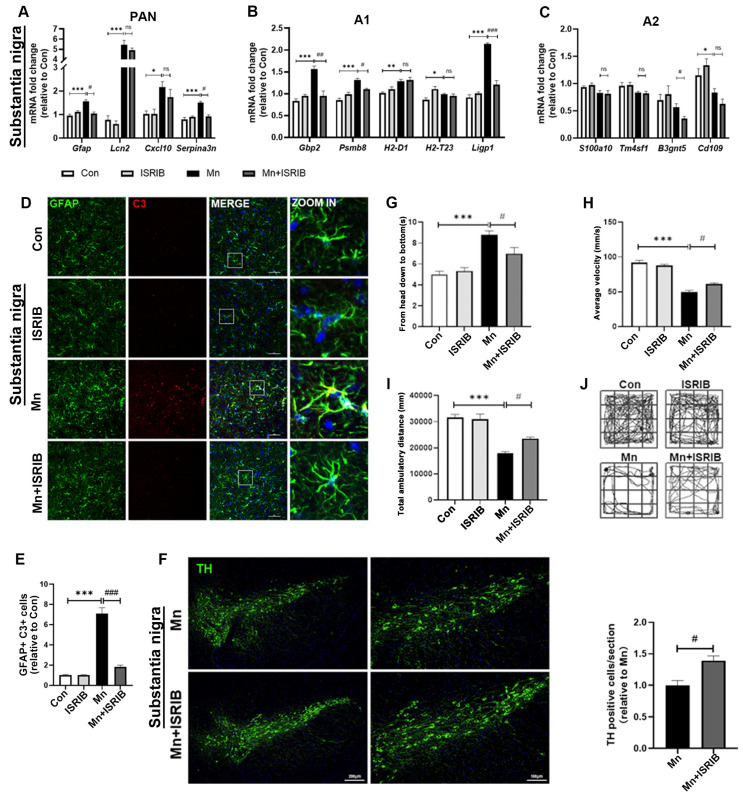
ISIRB alleviated Mn-induced neurotoxicity in mice. All experiments were divided into four groups: Con, Mn, ISRIB, and Mn + ISRIB group. (**A**–**C**) Bar charts showing relative expression of (**A**) PAN-specific, (**B**) A1-specific, and (**C**) A2-specific transcripts in the SN, analyzed by RT-qPCR (*n* = 3, * *p* < 0.05 vs. Con, ** *p* < 0.01 vs. Con, *** *p* < 0.001 vs. Con, ^#^ *p* < 0.05 vs. Mn, ^##^ *p* < 0.01 vs. Mn, ^###^ *p* < 0.001 vs. Mn, ns *p* > 0.05 vs. Mn). (**D**) Representative immunofluorescence staining of GFAP and C3 proteins in coronal sections of the SN after Mn exposure, as described in the experimental procedures. Labeling indicates GFAP (green) and C3 (red) proteins. The area within the white box is enlarged on the right. (**E**) Quantitative analysis of GFAP and C3 colocalization in immunofluorescence images (*** *p* < 0.001 vs. Con, ^###^ *p* < 0.001 vs. Mn). (**F**) Representative immunofluorescence staining of tyrosine hydroxylase (TH) in coronal sections of the SN after Mn exposure, as described in the experimental procedures. Scale bar: left, 200 μm; right, 100 μm. Quantification of TH fluorescence intensity was compared across treatment groups (^#^ *p* < 0.05 vs. Mn). (**G**) Pole test: time required for mice to climb down the pole (*n* = 10, *** *p* < 0.001 vs. Con, ^#^ *p* < 0.05 vs. Mn). (**H**–**J**) Open-field test of locomotor activity after Mn exposure: (**H**) representative movement traces, (**I**) average walking velocity, and (**J**) total ambulatory distance traveled (*n* = 10, *** *p* < 0.001 vs. Con, ^#^ *p* < 0.05 vs. Mn).

To elucidate the pivotal role of PERK pathway activation in Mn-induced A1 astrocyte transformation, we conducted two in vivo experiments involving PERK pathway modulation. First, we administered ISRIB via intraperitoneal injection to pharmacologically inhibit PERK signaling (Figure S6A). To evaluate the impact of PERK inhibition by ISRIB following Mn exposure, we measured the mRNA expression of PAN-specific, A1-specific, and A2-specific markers in the SN using RT-qPCR (Figure 6A–C). The experimental groups included: control (Con), Mn-treated, Con + ISRIB treated, and Mn + ISRIB treated groups. Compared with the Mn group, the Mn + ISRIB group showed significantly reduced mRNA expression of A1-specific markers (*Gbp2*, *Ligp1*, and *Psmb8*) (Figure 6B, *p* < 0.01). No significant changes were detected in the expression of pan-reactive or A2-specific markers in either the Mn or Mn + ISRIB groups (Figure 6A,C).

We also assessed the mRNA expression of these markers in striatum (Figure S6B–D). Transcriptional profiling revealed consistent results between the striatum and SN, demonstrating that pharmacological inhibition of the PERK signaling pathway mitigates A1-reactive astrocyte polarization following Mn exposure. Next, we used S100β and GFAP to label astrocytes in the SN (Figure 6D) and striatum (Figure S6E). Compared with Mn-treated mice, astrocytes from the Mn + ISRIB group displayed reduced soma size, thinner and less elaborate processes, and a significant decrease in C3-positive cell counts (Figure 6D). The proportion of A1 astrocytes, identified as C3^+^GFAP^+^ double-positive cells and S100β^+^C3^+^ double-positive cells, was also significantly reduced in the SN and striatum of Mn + ISRIB-treated mice (Figure 6D and Figure S6E).

Meanwhile, to evaluate neuronal dysfunction in other basal ganglia nuclei affected by Mn, anatomically registered cryosections of the basal ganglia were analyzed to quantify dopaminergic neurons in the striatum and SN. Compared with the control group, Mn exposure led to a significant reduction in synapse number in primary neurons (Figure 6E and Figure S6F, *p* < 0.01). Representative montage images of sections immunolabeled with anti-TH (green) are shown. TH immunohistochemistry revealed a significant increase in nigrostriatal dopaminergic neurons in Mn + ISRIB-treated mice compared with Mn-exposed mice, indicating that PERK pathway inhibition attenuates Mn-induced dopaminergic neurotoxicity in the SN and striatum (Figure 6E and Figure S6F). To determine whether PERK inhibition ameliorates Mn-induced motor deficits, we quantitatively assessed motor coordination and balance using standardized open-field behavioral paradigms. Mn + ISRIB-treated mice exhibited significantly improved locomotor performance compared with Mn-exposed mice, including increased movement distance and velocity in the open-field test (Figure 6F–H, *p* < 0.01). In summary, these findings underscore the role of the PERK in mediating Mn-induced A1 astrocyte transformation, dopaminergic neurotoxicity, and motor coordination dysfunction.

## 4. Discussion

Herein, we report that the PERK pathway contributes to Mn exposure-induced neurotoxicity by mediating ER stress in A1 reactive astrocytes. Although excessive-Mn-inhalation-induced neurotoxicity has been documented in multiple studies, the underlying mechanisms remain incompletely understood. Our previous studies demonstrated that Mn causes both direct toxicity on dopaminergic neurons and indirect toxicity mediated by microglial activation. Nevertheless, the potential involvement of other glial cells in this process has not been fully elucidated. Recent studies indicates that astrocytes, the most abundant glial cells in the CNS, are not only essential for maintaining homeostasis but are critically involved in various pathological conditions [25]. Current research on Mn poisoning has primarily focused on the dopaminergic nervous system, with numerous studies confirming an association between elevated Mn levels in the brain and impaired dopaminergic neurotransmission [26,27,28]. Mn preferentially accumulates in the basal ganglia, a dopamine (DA)-rich region [1]. Our in vitro results show that Mn-induced A1 activation damages neuronal synaptic structures without causing neuronal apoptosis. However, we observed that A1 astrocyte activation led to neuronal death in vivo. In the actual environment, astrocytes and neurons form synapses together, allowing A1 activation to directly affect neurons and produce more significant damage. In contrast, our in vitro experiments primarily involved collecting substances secreted by astrocytes into the culture medium, rather than allowing direct astrocyte–neuron interactions. Moreover, Mn-induced alternations in striatal DA content can lead to motor deficits [29]. Consistent with our previous studies, we observed that Mn exposure led to motor impairments and reduced TH expression in the SN and striatum regions.

Neuroinflammation represents a significant mechanism underlying Mn-induced neurotoxicity. While previous studies on inflammatory processes have primarily focus on Mn’s effects on microglial cells [30,31,32], the scientific community has increasingly recognized the important inflammatory contributions of astrocytes. Astrocytes can differentiate into either the A1 or A2 phenotype in response to diverse stimuli, indicating that their functional significance exceeds previous estimations. To further explore this phenomenon, we established in vivo and in vitro Mn exposure models to assess astrocytic responses. Real-time PCR and immunostaining analyses demonstrated that Mn exposure significantly upregulated A1-specific markers in Mn-targeted brain regions. In this study, we employed 100 mg/kg MnCl_2_ to establish a Mn-exposure animal model for investigating and confirming Mn’s neurotoxic effects. Our research specifically addresses occupational Mn exposure, which typically involve high-dose Mn or Mn compounds over short time periods. Our findings demonstrate that Mn exposure not only induces neurotoxicity but also activates astrocytes. Based on our previous studies, we confirmed that the 100 mg/kg MnCl_2_ dose is appropriate for establishing acute and sub-acute Mn exposure models [3,7]. However, whether chronic Mn exposure also triggers astrocytic A1 activation remains unclear, requiring further experimental investigation. To exclude the potential influence of microglia, primary astrocytes cultures were used to verify the direct effects of Mn exposure. The results demonstrated that Mn exposure directly induced the conversion astrocytes to the A1 phenotype. Notably, in our in vitro experiments, treatment with astrocyte-conditioned medium (ACM) did not induce neuronal apoptosis but significantly reduced synaptic density. Collectively, these findings suggest that Mn-induced neurotoxicity is mediated, at least in part, by A1-type reactive astrocytes.

The mechanisms underlying Mn-induced neurotoxicity are multifaceted. Our previous studies have demonstrated that several molecular pathways contribute to Mn neurotoxicity, including LRRK2-mediated autophagic dysfunction, NLRP3-cGAS/STING/NF-κB signaling, and miR-125b-2-3p/TFR1-mediated ferroptosis [3,5,33]. ER stress plays a critical role in the cellular stress responses, and numerous studies have established its involvement in astrocytic transformation [34]. To examine Mn-induced ER stress we performed transmission electron microscopy (TEM) and confocal imaging analyses, which revealed pronounced swelling of the ER in activated astrocytes following Mn exposure. These morphological changes strongly suggest that ER stress may be implicated in the activation of the A1 astrocytic phenotype. The UPR represents a critical cellular mechanism for responding to ER stress. When unfolded or misfolded proteins accumulate within the ER, the UPR is activated to restore normal ER function [35]. The PERK pathway constitutes a crucial branch of the UPR, a cellular stress response triggered by the accumulation of unfolded or misfolded proteins in the ER. Our previous research confirmed that the PERK pathway mediates Mn-induced neuroinflammation in microglia [32], but whether PERK mediates Mn-induced A1 astrocytic activation remained unclear. Our data show that the EIF2α-PERK signaling is activated in Mn-exposed astrocytes, indicating that PERK contributes to Mn-induced A1 astrocytic activation.

Recent studies have demonstrated that the inhibition of A1 astrocyte activation represents a promising therapeutic strategy for CNS disorders [36,37,38,39]. Glucagon-like peptide-1 receptor (GLP1R) agonists have been reported to alleviate PD symptoms by suppressing A1 astrocyte activation [40]. Additionally, fluoxetine, a selective serotonin reuptake inhibitor, has been shown to attenuate A1 astrocytic activation and consequently reduce neuropsychiatric manifestations [41]. During ER stress, PERK undergoes activation, subsequently phosphorylating EIF2α, which inhibits protein translation and thereby reduces the burden on the ER [42]. Modulation of ER stress and the UPR through the use of chemical chaperones has emerged as a potential therapeutic approach [43]. GSK2606414, a selective PERK inhibitor, blocked EIF2α-ATF4 signaling and mitigated ER stress [44]. Integrated stress response inhibitor B (ISRIB) inhibits the integrated stress response (ISR) by enhancing EIF2α nucleotide exchange activity, thereby restoring protein translation [45]. ISRIB has been shown to ameliorate vascular calcification (VC) pathogenesis by blocking EIF2α-ATF4 signaling, suggesting a potential new therapeutic target for VC prevention and treatment [46]. GSK2606414 is a specific PERK inhibitor that inhibits ER stress mediated by the EIF2α-ATF4 pathway. Our experiments revealed that while GSK2606414 effectively inhibits ER stress in vitro, its application in vivo faces significant limitations. First, when administered orally or intraperitoneally, GSK2606414 cannot penetrate the blood–brain barrier (BBB). Second, direct stereotactic injection into the brain caused severe adverse effects, including seizures, distress, and mortality in mice. We therefore switched to ISRIB, which can also block EIF2α-ATF4 signaling while crossing the BBB. Also, ISRIB has been considered a promising therapeutic candidate for Alzheimer’s disease (AD) [47]. Based on these beneficial properties and its regulatory effects on ER stress in the CNS, we investigated the role of ISRIB in Mn-induced A1 astrocytic activation. Our results demonstrated that ISRIB pretreatment effectively attenuated Mn-induced ER stress and A1 activation. Furthermore, neurobehavioral assessments revealed that ISRIB intervention significantly alleviated Mn-induced neurotoxicity, suggesting its potential therapeutic implications.

## 5. Limitations

Our research employed manganese exposure models to investigate the inducible effects of Mn on A1 astrocyte activation and ER stress mediated by the EIF2α-PERK pathway. Although we utilized both in vivo and in vitro experimental approaches, additional ER stress inhibition experiments should be conducted to further validate our findings. Furthermore, the mechanisms by which ATF6 and XBP1 contribute to A1 activation or Mn-induced neurotoxicity require further elucidation in subsequent studies.

## Data Availability

All data that support the findings of this study are included in this manuscript and its Supplementary Materials files.

## Supplementary Materials

The following supporting information can be downloaded at: https://www.mdpi.com/article/10.3390/toxics13110910/s1, Figure S1: Mn caused neurotoxicity in mice; Figure S2: Exposure to manganese leads to the activation of astrocytes in the mouse brain’s striatum; Figure S3: TEM representative images of the ultrastructural changes in striatum astrocytes; Figure S4: The protein level of p-PERK/PERK and EIF2α/p-EIF2α in the striatum after Mn exposure; Figure S5: Inhibition of PERK reduced Mn-induced proinflammatory response in vitro; Figure S6: ISIRB alleviated manganese exposure-induced damage to striatal neurons.
