# Supplementary material for: The EIF2α-PERK Signaling Pathway Mediates Manganese Exposure-Induced A1-Type Astrocytes Activation via Endoplasmic Reticulum Stress
Source: Toxics. 2025 Oct 23;13(11):910. doi: 10.3390/toxics13110910 (PMC12656517; doi:10.3390/toxics13110910)
Supplement: Supplementary file 1 [file toxics-13-00910-s001.zip › toxics-3861234-supplementary.pdf]

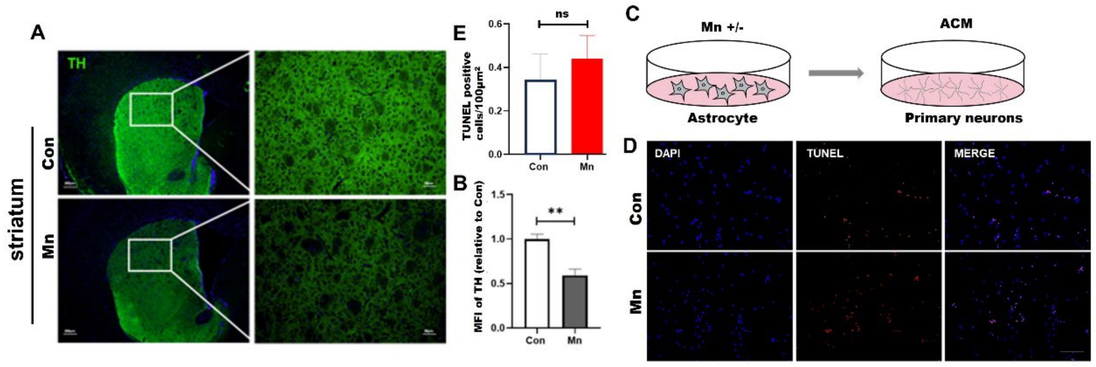

Fig. S1. Mn caused neurotoxicity in mice. (A) After Mn exposure, coronal sections of the striatum were immunostained for TH proteins by IHC as described in the Experimental procedures section, Scale bar = 25 µm. (B) TH fluorescence intensities were compared among different treatment groups. ***p* < 0.01. (C) Schematic diagram of Mn - stimulated astrocyte cultures that had developed A1 activation were transferred to neuronal medium for co – culture, ACM: Astrocyte conditioned medium. (D) Fluorescent staining image of primary neurons. DAPI marks all primary neurons, while TUNEL marks dead primary neurons. Scale bar = 50 µm. (E) Cell death rate statistics chart; n = 3, ns *p* > 0.05 vs. Con.

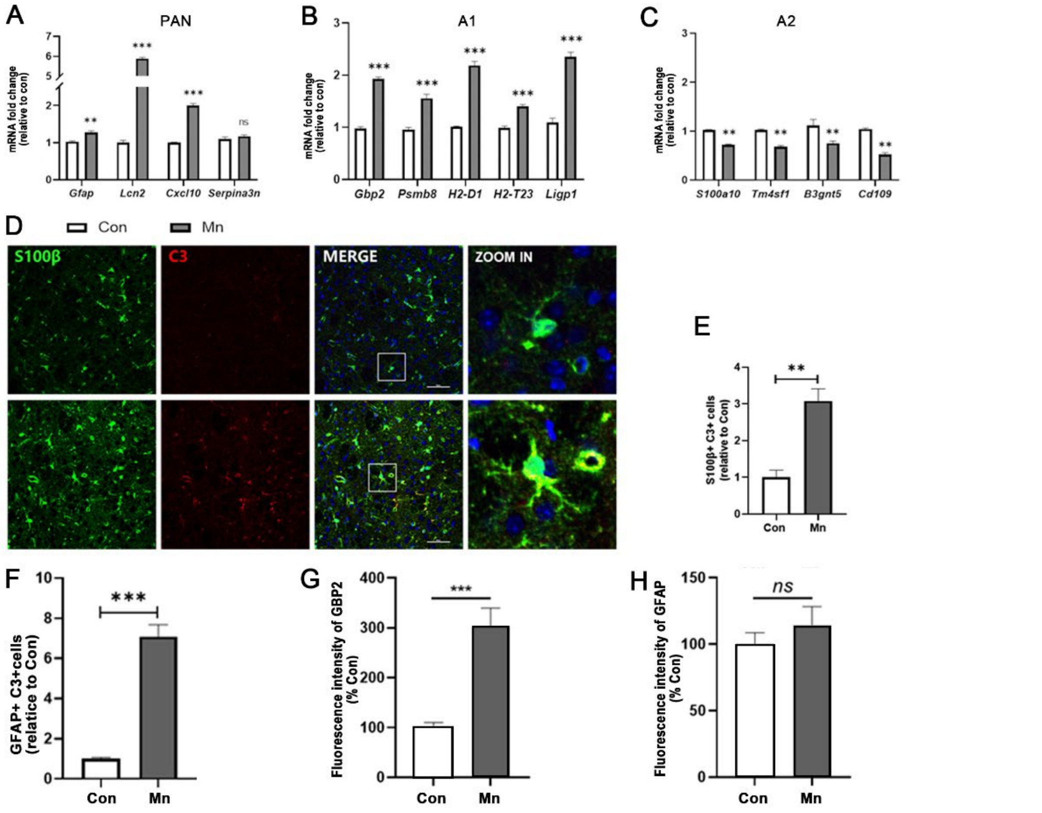

Fig. S2. Exposure to manganese leads to the activation of astrocytes in the mouse brain's striatum. (A-C) The mRNA levels of genes that functioned for PAN/A1/A2-special transcripts in the striatum exposed of Mn. **$p< 0.01$ *vs*. Con， ***$p< 0.001$ *vs*. Con.(D) Representative images of immunostaining (red for C3, green for S100β and blue for DAPI) in the striatum. (E) Immunohistochemical staining of S100β+ and C3+ astrocyte in the striatum. **$p < 0.01$, compared with the controls, n=3, Scale bar = 50 µm. (F) Immunohistochemical staining of GFAP+ C3+ astrocyte in the primary astrocytes. ***$p < 0.001$, compared with the controls, n=3. (G) Immunohistochemical staining of GBP2+ astrocyte in the primary astrocytes. ***$p < 0.001$, compared with the controls, n=3. (H) Immunohistochemical staining of GFAP astrocyte in the primary astrocytes. ns>0.05, compared with the controls, n=3.

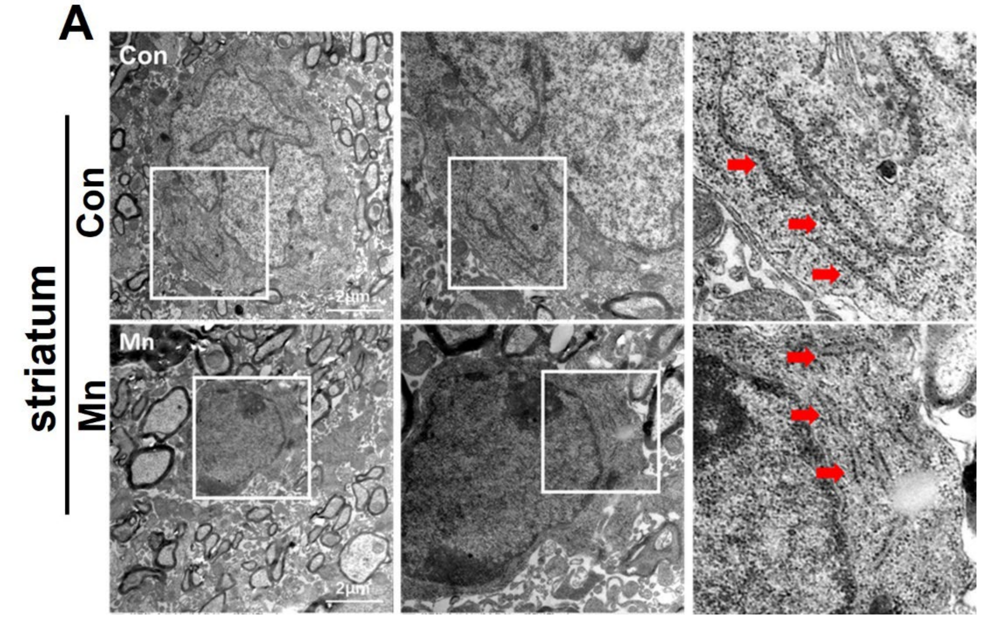

Fig. S3 TEM representative images of the ultrastructural changes in starium astrocytes (red arrowhead for ER). Scale bar in left side, 2 μm; Scale bar in right side, 1 μm.

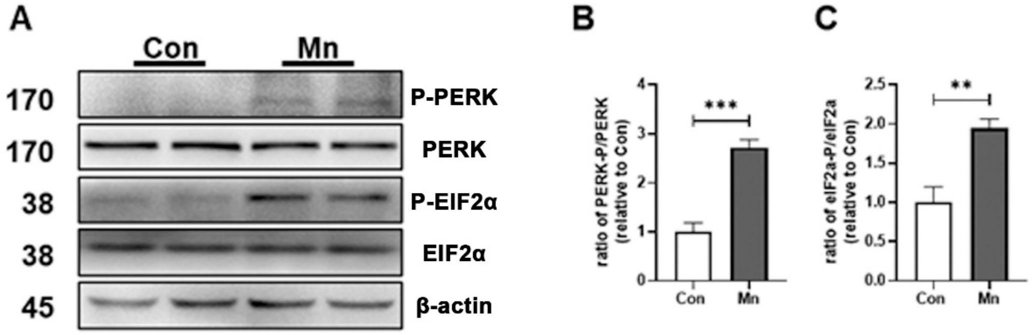

Fig. S4 The protein level of p-PERK/PERK and EIF2α/p- EIF2α in the striatum after Mn exposure. (A) The protein expressions of p-PERK, PERK, p-EIF2α and EIF2α were determined by Western blotting. (B) Gray scale histogram analysis of P-PERK/PERK. n=4, ****p*<0 .001, compared to the Con group. (C) Gray scale histogram analysis of P-EIF2α/EIF2αData are presented as mean SEM, n=4, ***p*< 0.01, compared to the Con group.

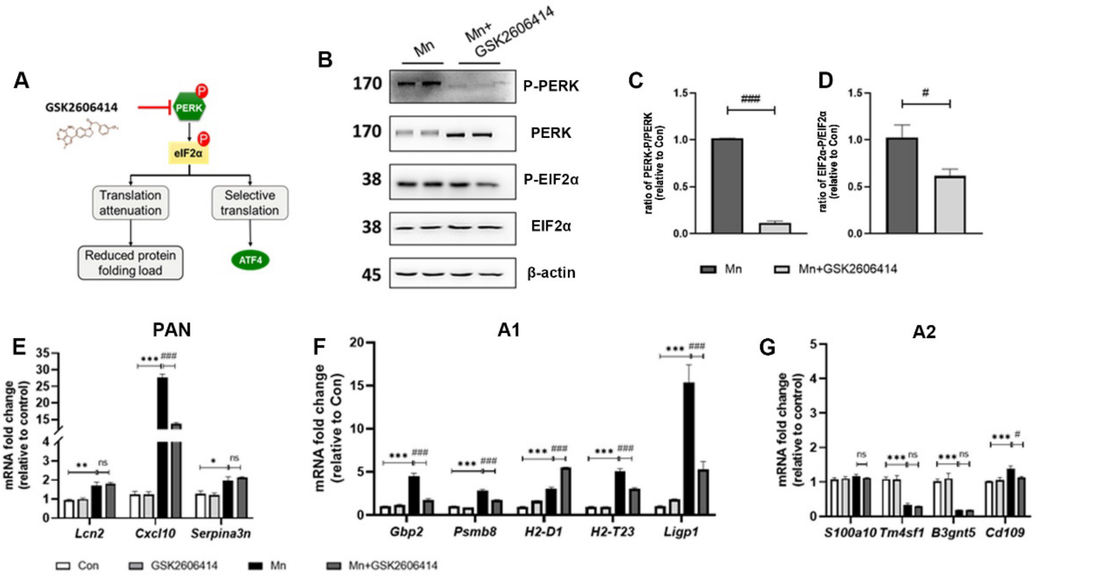

Fig. S5 Inhibition of PERK reduced Mn-induced proinflammatory response *in vitro*.

(A) Schematic diagram of GSK2606414 inhibition of the PERK pathway,(B-D) Following PERK inhibitors GSK2606414 pre-treatment and Mn exposure, Mn and Mn+GSK2606414 groups were analyzed for PERK/PERK(B) and EIF2α/EIF2α(D) protein levels by western blotting. (E-G) The bar chart of PAN-special (E), A1-special(F) and A2-special (G) transcripts in the primary astrocyte as analyzed by RT-qPCR. The experiment was divided into four groups: Con, GSK2606414, Mn and Mn+ GSK2606414, n = 3, *$p < 0.05$ *vs*. Con, **$p < 0.01$ *vs*. Con, ***$p < 0.001$ *vs*. Con, $^{\#}p < 0.05$ *vs*. Mn, $^{\#\#\#}p < 0.01$ *vs*. Mn, ns $p > 0.05$ *vs*. Mn.

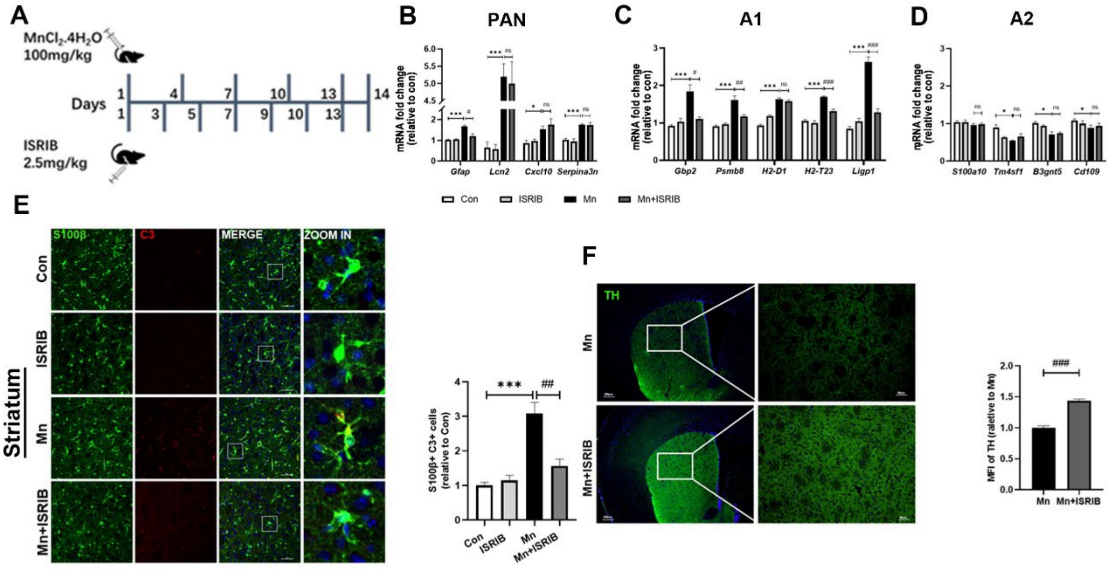

Fig. S6 ISIRB alleviated manganese exposure-induced damage to striatal neurons. (A) Experimental schematic on Mn treatment in mice. (B-C) The bar chart of PAN-special (B) A1-special(C) and A2-special, (D) transcripts in the striatum as analyzed by RT-qPCR. The experiment was divided into four groups: Con, ISRIB, Mn and Mn+ISRIB. (E) After Mn exposure, coronal sections of the striatum were immunostained for S100 β and C3 proteins by IHC as described in the Experimental procedures section, n=3, $^{***}P < 0.001$ *vs*. Con, $^{\#\#}P < 0.01$ *vs*. Mn. (F) After Mn exposure, coronal sections of the striatum were immunostained for TH proteins by IHC as described in the Experimental procedures section, left scale bar = 200 μm, right scale bar = 50 μm, TH fluorescence intensities were compared among different treatment groups, $^{\#\#\#}P < 0.001$, compared with the controls, n=3.
